# Population Frequencies of VWD-Associated Genotypes: Cross-Resource Evidence from RGC-ME and gnomAD

**DOI:** 10.3390/ijms27198866

**Published:** 2026-10-05

**Authors:** Giovanna D’Andrea, Maria d’Apolito, Maurizio Margaglione, Rosa Santacroce

**Affiliations:** Medical Genetics, Department of Clinical and Experimental Medicine, University of Foggia, 71122 Foggia, Italy; giovanna.dandrea@unifg.it (G.D.); maria.dapolito@unifg.it (M.d.); rosa.santacroce@unifg.it (R.S.)

**Keywords:** von Willebrand disease, population genomics, genotype frequency, penetrance, clinical registries

## Abstract

Population-scale sequencing can quantify genotypes associated with von Willebrand disease (VWD), but these frequencies cannot be translated directly into clinically manifest disease without phenotype and penetrance data. We therefore estimated subtype-specific VWD-associated genotype frequencies in RGC-ME, compared them with gnomAD, and examined which analytical assumptions limit clinical interpretation. Germline VWF variants classified in ClinVar as pathogenic or likely pathogenic (P/LP) on 1 August 2026 were normalized to GRCh38 and matched exactly to both resources. Subtypes were assigned only from unambiguous ClinVar phenotypes, and expected genotype frequencies were calculated from cumulative allele frequencies under dominant or recessive models. ClinVar contained 459 unique sequence-level P/LP alleles; 116 were shared by RGC-ME and gnomAD, 20 occurred only in RGC-ME, 68 only in gnomAD, and 255 in neither extract. The expected RGC-ME genotype frequencies per million individuals were 198, 16, 43, and 224 for types 1, 2A, 2B, and 2M, respectively, and 22.88 and 0.008 for types 2N and 3. Corresponding RGC-ME/gnomAD ratios ranged from 0.753 to 0.911. Shared allele frequencies were strongly concordant (Pearson r = 0.933; Spearman ρ = 0.887), although 238/459 alleles lacked an unambiguous subtype assignment. The cumulative frequency of P/LP VWF alleles was similar across resources, whereas subtype-specific expected genotype frequencies varied with phenotype annotation, the contribution of recurrent alleles, inheritance assumptions, and incomplete variant capture. Population databases can therefore quantify allele burden and model the frequency of VWD-associated genotypes, but clinical registries are required to determine penetrance, genotype–phenotype relationships, and the prevalence of clinically diagnosed VWD.

## 1. Introduction

Von Willebrand disease (VWD) is the most common inherited bleeding disorder and results from quantitative or qualitative defects of von Willebrand factor (VWF), a multimeric glycoprotein that stabilizes factor VIII and initiates platelet adhesion through binding of VWF to GPIbα within the platelet GPIb-V-IX complex [1,2,3,4,5]. Current classification distinguishes partial quantitative deficiency (type 1), virtual absence of VWF (type 3), and qualitative defects (types 2A, 2B, 2M, and 2N) [2,3]. Type 1 usually shows autosomal dominant inheritance with variable penetrance; types 2A, 2B, and 2M are also predominantly dominant, whereas types 2N and 3 are generally recessive. Type 2B is mechanistically distinct because gain-of-function variants increase VWF binding to platelet GPIbα [3,4,6,7,8,9,10,11,12].

Reported prevalence varies markedly with ascertainment. Population screening identifies low VWF levels or bleeding phenotypes suggestive of VWD in approximately 0.6–1.3% of individuals, whereas clinically recognized VWD is estimated at several tens per 100,000 and severe type 3 disease at approximately 0.1–5.3 per million [13,14,15,16,17,18]. This gap reflects incomplete penetrance, ABO effects, age- and stress-dependent VWF levels, referral patterns and the distinction between a biochemical trait and clinically relevant bleeding [4,14,19,20,21,22,23,24].

Large population sequencing datasets can quantify the population frequency of VWD-associated genotypes by aggregating disease-associated alleles [25,26]. Interpretation requires an explicit causal allele set, phenotype-specific annotation, an appropriate inheritance model, and evidence that the resulting burden is reproducible across different datasets. Even when reproducible, genotype frequency does not establish clinical prevalence because penetrance and diagnostic phenotype remain unknown [8,9,27,28,29].

The six major VWD phenotypes were recently examined in gnomAD v4.1 [30]. We independently calculated expected genotype frequencies from the RGC Million Exome (RGC-ME) catalogue, which contains 983,578 exomes from multiple ancestry groups [31], and then used the published gnomAD genotype-frequency values as a cross-dataset benchmark. The datasets are large but are not population-representative surveys: their cohort composition, ascertainment, ancestry balance, and available metadata differ. In addition, both contain UK Biobank-derived data, so the comparison is not a replication in fully independent populations. Our aims were to quantify cross-dataset reproducibility, identify sources of discordance, and define what additional clinical evidence is needed before population allele frequencies can inform clinically manifest VWD prevalence.

## 2. Results

### 2.1. Conservative Subtype-Specific VWD-Associated Genotype Frequencies

The ClinVar download contained 540 VWF P/LP records. Of these, 459 were unique sequence-level alleles suitable for exact comparison; 81 were structural, copy-number, or otherwise unresolved records. Conservative subtype assignment yielded 65 alleles for type 1, 22 for type 2A, 15 for type 2B, 12 for type 2M, 10 for type 2N, and 97 for type 3. The model-derived expected genotype frequencies in RGC-ME were 198, 16, 43, 224, 22.88, and 0.008 per million for types 1, 2A, 2B, 2M, 2N, and 3, respectively (Table 1). These values are the primary subtype-specific expected genotype frequencies.

Dominant models use 2*q* − *q*^2^; type 2N and type 3 use *q*^2^. The reported values are model-derived expected genotype frequencies, not observed case counts or penetrance-adjusted clinical prevalence. Alleles with generic or multiple conditions were not forced into a subtype.

Corresponding RGC-ME/gnomAD genotype-frequency ratios were 0.782, 0.753, 0.859, 0.782, 0.911, and 0.762 for types 1, 2A, 2B, 2M, 2N, and 3, respectively. Among 116 shared P/LP alleles, frequencies were highly concordant (Pearson r = 0.933 after log transformation; Spearman ρ = 0.887; Supplementary Figure S1). The cumulative P/LP allele frequency was 0.006353 in gnomAD and 0.005931 in RGC-ME (ratio 0.934). Restricting the analysis to alleles with at least two ClinVar review stars showed a similar concordance between the two datasets (Pearson r = 0.940; Spearman ρ = 0.939).

### 2.2. Comparison with Published gnomAD Genotype-Frequency Calculations

When the broader phenotype sets [30] were applied to RGC-ME, the expected genotype frequencies for type 1 were similar across datasets (10.61 versus 10.13 per 1000). The expected RGC-ME genotype frequency for type 2A was 770 per million, corresponding to 59.8% of the published gnomAD value, whereas the corresponding values for types 2B and 2M were 19.9% and 10.8%, respectively. For the recessive model, the corrected expected biallelic genotype frequencies were 29.29 per million for type 2N and 0.84 per million for type 3, corresponding to 86.3% and 63.0% of the respective gnomAD values (Table 2). These exploratory upper-range calculations reproduce the phenotype assignments of the published allele set and should be distinguished from the primary, independently curated genotype-frequency calculations.

### 2.3. Contribution of Recurrent Variants

The number of variants assigned to a phenotype did not indicate whether the corresponding expected genotype frequency was strongly influenced by a recurrent variant. The most frequent variant contributed 64.7% of the cumulative allele frequency used for the type 1 calculation, 29.2% for type 2A, 78.5% for type 2B, 70.8% for type 2M, and 85.1% for type 2N (Table 3).

The type 2A calculation was less concentrated in a single variant than those for the other dominant subtypes. Type 3 also differed because no single variant contributed more than 11.8%, and its cumulative allele burden was distributed across many rare variants. For type 1, the similar overall values across datasets were partly attributable to the comparable frequency of the recurrent p.Tyr1584Cys variant. This numerical contribution should not be interpreted as indicating that p.Tyr1584Cys is representative of type 1 VWD, which is genetically and clinically heterogeneous. More generally, when a calculation is strongly influenced by one recurrent allele, differences in that allele’s frequency or classification can substantially affect the resulting model-derived genotype frequency.

### 2.4. Effect of Variant Selection

When the analysis was restricted to variants currently classified in ClinVar as pathogenic or likely pathogenic, 7.4% of the original type 1 expected genotype frequency was retained, together with 6.9% for type 2A, 11.9% for type 2B, 15.7% for type 2M, and 16.8% for type 3 (Table 4). In contrast, 88.1% of the type 2N value was retained because p.Arg854Gln, which contributed most to its cumulative allele frequency, has a stable pathogenic classification. Thus, the model-derived genotype frequencies were affected more by the choice of included variants than by the population database used. Although these calculations are not definitive because ClinVar coverage and classifications are incomplete and may change, they indicate that variant selection introduced more uncertainty than sampling or differences between the two datasets.

Table 5 compares the expected genotype frequencies calculated using the conservative and broader variant sets. The higher values would correspond to clinical frequency only if all included genotypes had complete penetrance. The values from the conservative analysis should not be considered lower limits because some affected individuals may carry variants that were excluded or not detected.

### 2.5. Review of the Published Genotype-Frequency Calculations

We identified 321 variant entries across the VWD phenotypes. One type 3 variant, p.Asp2509LeufsTer23 (c.7524_7525del; rs1591836930), was listed twice because of a page break, leaving 320 unique variants. All subsequent analyses included each allele only once. Of the 320 unique variants, 197 had an exact DNA-level match in RGC-ME. Another 12 had the same rsID but a different nucleotide change at the same position. These included four type 1, one type 2B, one type 2M, and six type 3 variants. The largest difference involved c.7080C>A (p.Cys2360Ter), which corresponds to a genomic G > T change, whereas RGC-ME contained a G > A change at the same position. Counting the G > A allele would therefore assign the frequency of one variant to another. These 12 mismatches explained the differences in the RGC-ME expected genotype-frequency calculations.

## 3. Discussion

The present analysis showed that the gene-wide ClinVar P/LP variant set produced similar cumulative VWF allele frequencies in RGC-ME and gnomAD (ratio, 0.934), with a strong correlation between the frequencies of shared alleles. Agreement was lower and more variable for the model-derived subtype-specific genotype frequencies (ratios, 0.753–0.911), which were more affected by variant classification and the numerical contribution of a few recurrent alleles. The conservative and broader analyses address different questions. The conservative analysis includes P/LP variants clearly assigned to a VWD subtype, whereas the broader analysis also includes variants with less certain pathogenicity or subtype assignment. The difference between the results indicates that variant selection affected the calculated genotype frequencies more than sampling differences between the two datasets. In the broader published allele sets, much of the calculated burden for types 2B and 2M was numerically attributable to p.Pro1266Leu and p.Pro1266Gln, respectively; neither exact allele was identified in RGC-ME. This explains part of the cross-dataset discrepancy but does not imply that either variant is representative of the corresponding subtype. p.Pro1266Leu, previously reported as p.Pro503Leu, increases VWF-GPIbα binding and is associated with the Malmö/New York phenotype, increased ristocetin-induced platelet agglutination, preserved multimers, and usually mild bleeding without thrombocytopenia [32,33,34,35]. p.Pro1266Gln has been classified as both type 2B and type 2M [35,36,37], illustrating the need for allele-specific functional and phenotypic evidence. The closer agreement for types 1 and 2N was likewise partly influenced by similar frequencies of the recurrent p.Tyr1584Cys and p.Arg854Gln alleles. Nevertheless, type 1 VWD is highly heterogeneous, and no single recurrent variant should be considered its main or representative variant.

Clinical cohorts provide complementary evidence that is not available from population databases. In a German cohort of patients with type 2 VWD, VWF variants were identified in 187 of 196 index cases, including 45 previously unreported variants. Another German cohort of patients with quantitative VWD showed marked genetic heterogeneity, copy-number variants, and different variant detection rates in types 1 and 3 VWD [38,39]. These studies show that clinically confirmed VWD includes rare and technically complex variants that may be absent or underrepresented in general-population datasets. They also show that the frequency of p.Pro1266 alleles alone cannot determine their subtype or clinical effects. Disease-specific databases can address this gap by linking variants to clinical cases, laboratory findings, and curated evidence. EAHAD/LOVD and HGMD complement ClinVar but were not used as primary sources in this analysis because they differ in scope, access, classification criteria, and case-based denominators [40,41]. Directly comparing variant counts across these databases would therefore not show how many variants are missed by population sequencing. Future studies should compare exact alleles across these resources and validate them in patient registries containing well-documented clinical and laboratory data.

The genetic data provide model-derived expected frequencies of VWD-associated genotypes but do not measure the prevalence of symptomatic VWD. Population databases lack the bleeding history, VWF and FVIII measurements, ABO blood group, age, and longitudinal data needed to estimate penetrance [19,20,22,23,24,42,43,44]. With complete penetrance, an expected genotype frequency could approximate clinical frequency; with incomplete penetrance, it would overestimate it. However, these data do not provide a formal lower bound for clinical frequency. Clinical interpretation should therefore remain based on phenotype. A variant carrier may have borderline VWF levels with little or no bleeding, whereas a patient with VWD may carry a variant that is absent from ClinVar or not detected by exome sequencing. Diagnosis and management should continue to consider bleeding history, laboratory findings, and VWD subtype, particularly before surgery or pregnancy [2,44,45,46]. Similar genotype–phenotype variability has also been reported in studies of incidental findings in clinical exomes and in other inherited bleeding disorders [47,48].

Under the assumption of random mating, *q*^2^ estimates the expected frequency of individuals carrying two included pathogenic or likely pathogenic alleles for the recessive forms, types 2N and 3. This calculation does not confirm that the two alleles are located on different copies of VWF or that they form a disease-causing combination. It may also miss deletions, other structural variants, and deep intronic variants, leading to underestimation of *q* and *q*^2^. Conversely, combining alleles that do not cause disease together may lead to overestimation. Population structure, consanguinity, and uncertain phase may affect the calculated frequency in either direction. These values therefore represent expected genotype frequencies, not observed numbers of patients.

Correct subtype classification remains clinically important. In type 2B VWD, laboratory and genetic testing can distinguish gain-of-function VWF variants from type 2A VWD and platelet-type VWD. This distinction guides treatment because desmopressin may worsen thrombocytopenia [2,9,10,11,33,35,45,49,50,51]. In type 2N VWD, VWF–FVIII binding assays or genetic testing can distinguish the condition from mild hemophilia A [2,52,53,54,55,56]. Diagnosing type 3 VWD requires the identification of two severe VWF defects, including structural variants that may be missed by exome sequencing [45,57].

This study has several limitations. RGC-ME and gnomAD both include UK Biobank data. Therefore, agreement between them reflects reproducibility across datasets rather than fully independent replication. The available RGC-ME extract did not include ancestry-specific, homozygote, or phenotype data. ClinVar subtype assignments and review status were incomplete, and we did not reassess every variant using the original clinical and functional evidence. Canonical splice-site variants were included when available in ClinVar and the exome datasets, but deep intronic and structural variants were not systematically captured. Finally, the expected genotype frequencies for recessive VWD assume random mating and that two included alleles can cause disease when inherited together. These limitations have a greater effect on biological interpretation than the reported sampling intervals.

## 4. Materials and Methods

### 4.1. Data Sources and Study Design

**Figure 1:**
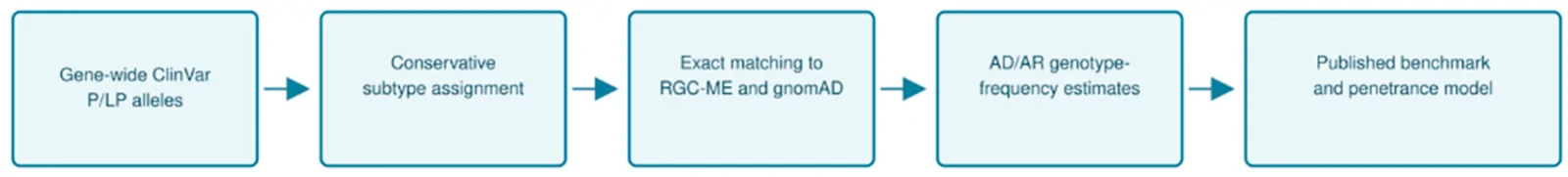
Study design. Gene-wide ClinVar P/LP alleles were used to calculate subtype-specific expected genotype frequencies in RGC-ME; gnomAD and the published study were subsequent cross-dataset comparators. AD, autosomal dominant; AR, autosomal recessive; P/LP, pathogenic/likely pathogenic.

This cross-sectional population-genomic study used an RGC-ME VWF extract containing 8108 PASS variants and a gnomAD v4.1 VWF extract containing 10,397 variants. RGC-ME provided global allele counts, allele numbers, and allele frequencies but no ancestry-specific, homozygote, or phenotype data in the supplied extract; gnomAD provided global and ancestry-specific counts and homozygote counts. Both are multi-cohort, multi-ancestry research datasets and were not designed to be representative samples of the general population. Their ancestry composition and ascertainment differ, and both include UK Biobank-derived data. Therefore, we describe the analysis as cross-dataset comparison, not validation in completely independent individuals. The workflow is summarized in Figure 1.

### 4.2. Independent Construction of Phenotype-Specific Variant Sets

The primary causal set was constructed without using the published mutation list. All VWF records were downloaded from ClinVar on 1 August 2026 [58], and germline classifications of pathogenic, likely pathogenic, or pathogenic/likely pathogenic were retained. Canonical Sequence–Position–Deletion–Insertion (SPDI) representations from ClinVar and the VCF-style alleles from RGC-ME and gnomAD were converted to a common minimal GRCh38 representation. Variants were considered matched only when they represented the same molecular allele. Deep intronic variants were not systematically evaluated because exome capture is incomplete outside targeted regions. Large copy-number variants or structural variants lacking a sequence-level SPDI representation were described separately because they could not be measured comparably in the supplied population extracts.

For the primary conservative analysis, ClinVar conditions were mapped to type 1, 2A, 2B, 2M, 2N, or 3 only when a single specific subtype was assigned. Generic VWD, unspecified type 2 VWD, records associated with multiple subtypes, and records without a specified VWD condition were included in the unassigned group. Review status ranged from zero-star submissions to expert-panel review. A sensitivity analysis restricted to alleles with at least two review stars assessed whether cross-resource frequency concordance persisted when variants supported by stronger evidence were considered. Because type 2B VWD is defined by increased VWF binding to platelet GPIbα, a ClinVar subtype label was considered an annotation rather than independent functional evidence.

### 4.3. Published gnomAD Genotype-Frequency Calculations as an External Comparator

After the independent RGC-ME expected genotype frequencies had been calculated, the phenotype-specific genotype-frequency values reported by Seidizadeh et al. [30] were used as an external gnomAD comparator. Supplementary Table S1 from that publication was extracted to investigate discrepancies and perform allele-level sensitivity analyses. Cross-dataset matching required identity of chromosome position, reference allele, and alternate allele; an rsID alone was not accepted as evidence of molecular identity [59]. Duplicate rows and instances in which the same rsID referred to different molecular alleles were recorded as secondary technical findings.

### 4.4. Genotype-Frequency Models

For cumulative subtype-associated allele frequency *q*, the dominant-model carrier frequency was estimated as 2*q* − *q*^2^ and the recessive-model biallelic frequency as *q*^2^. Types 1, 2A, 2B, and 2M were modelled as predominantly dominant; types 2N and 3 were modelled as recessive. The *q*^2^ approximation assumes random pairing of all qualifying alleles and cannot establish phase, compound-heterozygous compatibility, or detection of structural and deep intronic variants. Missing pathogenic alleles generally lower *q*^2^, whereas inclusion of mutually incompatible or incorrectly classified alleles can raise it. For type 2B, the calculation estimates carriers of annotated alleles; interpretation additionally requires evidence for the defining gain-of-function mechanism. All outputs are genotype-frequency estimates, not penetrance-adjusted clinical prevalence.

### 4.5. Cross-Database Transferability and Allelic Architecture

Cross-database reproducibility was assessed using the RGC-ME/gnomAD ratio of each phenotype-specific expected genotype frequency and Pearson and Spearman correlations among exactly matched alleles. Ratios for recessive phenotypes were therefore calculated from *q*^2^ rather than *q*. For each phenotype, we also quantified the contributions of the most frequent and three most frequent variants and performed leave-one-variant-out analyses. Agreement between databases was interpreted as reproducibility of the selected genetic burden, not as evidence of equivalent clinical prevalence or penetrance.

### 4.6. Evidence Levels, Ancestry, and Penetrance Scenarios

The primary analysis included current, gene-wide ClinVar P/LP alleles with an unambiguous subtype assignment. Sensitivity analyses considered the broader published allele set and applied progressively stricter evidence criteria; variants with conflicting or uncertain classifications were excluded from the most stringent evidence level. For the cross-database correlation analysis, the dataset was restricted to alleles with at least two ClinVar review stars. These analyses assessed the robustness of the results to differences in classification quality but did not constitute a de novo ACMG/AMP classification. gnomAD ancestry-specific allele frequencies were calculated from the allele count and allele number reported for each variant. Current ClinGen VWF-specific recommendations informed the interpretation, particularly for qualitative type 2 variants [60].

### 4.7. Clinical-Phenotype Prevalence Bounds

For each subtype, the expected genotype frequency was considered a potential upper estimate of clinical phenotype frequency only under complete penetrance, correct subtype assignment, and presence of the required genotype. The conservative P/LP calculation was not treated as a formal lower bound because the databases lack bleeding history, laboratory phenotype, and variant-specific penetrance. For recessive types 2N and 3, *q*^2^ further assumes random mating and functional compatibility among paired alleles; phase, structural variants, and compound-heterozygous combinations could not be assessed directly.

### 4.8. Uncertainty and Software

Sampling intervals for RGC-ME cumulative allele burdens were approximated from Poisson count intervals and propagated through the dominant or recessive model. These intervals quantify sampling error only and do not capture uncertainty in pathogenicity, penetrance, phase, or cohort overlap. Analyses were performed in Python v.3.12.14 using pandas, NumPy, SciPy, and pdfplumber. Values were retained at full precision and rounded only for presentation.

## 5. Conclusions

RGC-ME and gnomAD showed similar cumulative frequencies of VWD-associated P/LP alleles. However, the subtype-specific expected genotype frequencies depended on annotation quality, the contribution of recurrent variants, and inheritance assumptions. These datasets are therefore useful for quantifying population allele burden and modelling the expected frequency of VWD-associated genotypes, but not for determining the number of clinically affected patients. Reliable estimates of clinical prevalence and genotype–phenotype relationships require harmonized disease-specific databases and well-characterized patient registries containing bleeding history, laboratory findings, variant phase, structural variants, and penetrance data.

## Figures and Tables

**Table 1:** Gene-wide expected genotype frequencies based on ClinVar P/LP alleles with conservative phenotype assignment.

Phenotype	P/LP Alleles	Shared	RGC-Only	gnomAD Expected Genotype Frequency	RGC-ME Expected Genotype Frequency	RGC/gnomAD
Type 1	65	13	3	254/million	198/million	0.782
Type 2A	22	3	1	21/million	16/million	0.753
Type 2B	15	2	0	50/million	43/million	0.859
Type 2M	12	5	0	286/million	224/million	0.782
Type 2N	10	6	0	25.11/million	22.88/million	0.911
Type 3	97	21	3	0.0105/million	0.0080/million	0.762
Unassigned/ambiguous	238	66	13	Not interpretable	Not interpretable	—

**Table 2:** Expected genotype frequencies calculated using the published phenotype-specific allele sets.

Phenotype	Model	Phenotype Set, n	gnomAD Expected Genotype Frequency	RGC-ME Expected Genotype Frequency	Ratio
Type 1	AD	94	10,611/million	10,127/million	0.954
Type 2A	AD	43	1287/million	770/million	0.598
Type 2B	AD	12	1702/million	338/million	0.199
Type 2M	AD	25	1529/million	165/million	0.108
Type 2N	AR	130	33.948/million	29.289/million	0.863
Type 3	AR	153	1.337/million	0.843/million	0.630

AD, autosomal dominant; AR, autosomal recessive; n: number of phenotype-specific alleles. Values are model-derived expected genotype frequencies based on 320 unique source alleles and exact GRCh38 matching; they are not observed clinical prevalence.

**Table 3:** Contribution of recurrent variants to phenotype-specific allele burdens and expected genotype frequencies.

Phenotype	Most Frequent Variant	Contribution to Cumulative Allele Frequency	Top 3 Share	RGC/gnomAD	Interpretation
Type 1	p.Tyr1584Cys	64.7%	73.2%	0.954	Calculation strongly influenced by a recurrent allele shared by both resources
Type 2A	p.Gly1672Arg	29.2%	55.1%	0.598	Moderately distributed estimate
Type 2B	p.Pro1266Leu	78.5%	91.2%	0.199	Atypical GoF allele absent from RGC-ME
Type 2M	p.Pro1266Gln	70.8%	83.3%	0.108	Pro1266 requires subtype-specific review
Type 2N	p.Arg854Gln	85.1%	87.8%	0.863	Calculation strongly influenced by a recurrent pathogenic allele shared by both resources
Type 3	No single variant	11.8%	27.3%	0.630	Burden spread across many rare variants

**Table 4:** Sensitivity of expected genotype frequencies to variant-evidence criteria.

Phenotype	Published Expected Genotype Frequency	Curated P/LP Expected Genotype Frequency	Current ClinVar P/LP Expected Genotype Frequency	Retained
Type 1	10,611/million	936/million	784/million	7.4%
Type 2A	1287/million	261/million	89/million	6.9%
Type 2B	1702/million	253/million	202/million	11.9%
Type 2M	1529/million	257/million	240/million	15.7%
Type 2N	33.948/million	30.551/million	29.895/million	88.1%
Type 3	1.337/million	0.472/million	0.224/million	16.8%

P/LP, pathogenic/likely pathogenic. Curated P/LP includes variants classified as P/LP either in the source or current annotation; current ClinVar P/LP includes variants classified as P/LP in the ClinVar field of the supplied gnomAD extract, while retaining the phenotype assignments from the published allele set.

**Table 5:** Range of model-derived expected genotype frequencies in gnomAD and RGC-ME.

	Conservative Estimate		Broader Estimate	
Phenotype	gnomAD	RGC-ME	gnomAD	RGC-ME
Type 1	254/million	198/million	10,611/million	10,127/million
Type 2A	21/million	16/million	1287/million	770/million
Type 2B	50/million	43/million	1702/million	338/million
Type 2M	286/million	165/million	1529/million	224/million
Type 2N	25.11/million	22.88/million	33.948/million	29.289/million
Type 3	0.0105/million	0.0080/million	1.337/million	0.843/million

Values show the range of expected genotype frequencies obtained with the conservative and broader variant sets; they are not formal limits of clinical prevalence.

## Data Availability

The original contributions presented in this study are included in the Supplementary Materials. Further inquiries can be directed to the corresponding authors.

## Supplementary Materials

The following supporting information can be downloaded at: https://www.mdpi.com/article/10.3390/ijms27198866/s1.

## References

[1] Leebeek F.W.G., Eikenboom J.C.J. (2016). Von Willebrand’s disease. N. Engl. J. Med..

[2] James P.D., Connell N.T., Ameer B., Di Paola J., Eikenboom J., Giraud N., Haberichter S., Jacobs-Pratt V., Konkle B., McLintock C. (2021). ASH ISTH NHF WFH 2021 guidelines on the diagnosis of von Willebrand disease. Blood Adv..

[3] Sadler J.E., Budde U., Eikenboom J.C.J., Favaloro E.J., Hill F.G., Holmberg L., Ingerslev J., Lee C.A., Lillicrap D., Mannucci P.M. (2006). Update on the pathophysiology and classification of von Willebrand disease. J. Thromb. Haemost..

[4] Seidizadeh O., Peyvandi F., Mannucci P.M. (2024). Von Willebrand disease. Nat. Rev. Dis. Primers.

[5] Yan M., Wang Z., Qiu Z., Cui Y., Xiang Q. (2024). Platelet signaling in immune landscape: Comprehensive mechanism and clinical therapy. Biomark. Res..

[6] Castaman G., Federici A.B., Rodeghiero F., Mannucci P.M. (2003). Von Willebrand’s disease in the year 2003. Haematologica.

[7] Goodeve A.C. (2010). The genetic basis of von Willebrand disease. Blood Rev..

[8] Baronciani L., Goodeve A., Peyvandi F. (2017). Molecular diagnosis of von Willebrand disease. Haemophilia.

[9] Christopherson P.A., Haberichter S.L., Flood V.H. (2022). Genetic testing and the diagnosis of von Willebrand disease. J. Thromb. Haemost..

[10] Seidizadeh O., Baronciani L., Lillicrap D., Peyvandi F. (2024). Application of genetic testing for the diagnosis of von Willebrand disease. J. Thromb. Haemost..

[11] Seidizadeh O., Lillicrap D. (2026). Molecular genetic testing in von Willebrand disease: Past, present, and beyond. Haematologica.

[12] Keeney S., Cumming A.M. (2001). The molecular biology of von Willebrand disease. Clin. Lab. Haematol..

[13] Rodeghiero F., Castaman G., Dini E. (1987). Epidemiological investigation of the prevalence of von Willebrand’s disease. Blood.

[14] Werner E.J., Broxson E.H., Tucker E.L., Giroux D.S., Shults J., Abshire T.C. (1993). Prevalence of von Willebrand disease in children. J. Pediatr..

[15] Bowman M., Hopman W.M., Rapson D., Lillicrap D., Silva M., James P. (2010). A prospective evaluation of the prevalence of symptomatic von Willebrand disease. J. Thromb. Haemost..

[16] Corrales-Medina F.F., Federici A.B., Srivastava A., Dougall A., Millar C.M., Roberts J.C., Jaffray J., Berntorp E. (2023). Epidemiology and burden of von Willebrand disease: A systematic review. Blood Rev..

[17] Stonebraker J.S., Iorio A., Lavin M., Rezende S.M., Srivastava A., Pierce G.F., Coffin D., Tootoonchian E., Makris M. (2023). Global prevalence of von Willebrand disease. Haemophilia.

[18] Castaman G., Bramante Federici A. (2026). Von Willebrand disease: Classification and epidemiology. Haematologica.

[19] Gill J.C., Endres-Brooks J., Bauer P.J., Marks W.J., Montgomery R.R. (1987). The effect of ABO blood group on the diagnosis of von Willebrand disease. Blood.

[20] O’Donnell J., Laffan M.A. (2001). The relationship between ABO histo-blood group, factor VIII and von Willebrand factor. Transfus. Med..

[21] Sanders Y.V., Eikenboom J., de Wee E.M., van der Bom J.G., Cnossen M.H., Degenaar-Dujardin M.E.L., Fijnvandraat K., Kamphuisen P.W., Gorkom B.A.P.L., Meijer K. (2013). Reduced prevalence of arterial thrombosis in von Willebrand disease. J. Thromb. Haemost..

[22] Atiq F., Meijer K., Eikenboom J., Fijnvandraat K., Mauser-Bunschoten E.P., van Galen K.P.M., Nijziel M.R., Ypma P.F., de Meris J., Laros-van Gorkom B.A.P. (2018). Comorbidities associated with higher von Willebrand factor levels may explain age-related increase. Br. J. Haematol..

[23] James P.D., Lillicrap D. (2012). von Willebrand disease: Clinical and laboratory lessons learned from the large VWD studies. Am. J. Hematol..

[24] Swystun L.L., Lillicrap D. (2018). Genetic regulation of plasma von Willebrand factor levels in health and disease. J. Thromb. Haemost..

[25] Karczewski K.J., Francioli L.C., Tiao G., Cummings B.B., Alfoldi J., Wang Q., Collins R.L., Laricchia K.M., Ganna A., Birnbaum D.P. (2020). The mutational constraint spectrum quantified from variation in 141,456 humans. Nature.

[26] Chen S., Francioli L.C., Goodrich J.K., Collins R.L., Kanai M., Wang Q., Alföldi J., Watts N.A., Vittal C., Gauthier L.D. (2024). A genomic mutational constraint map using variation in 76,156 human genomes. Nature.

[27] Richards S., Aziz N., Bale S., Bick D., Das S., Gastier-Foster J., Grody W.W., Hegde M., Lyon E., Spector E. (2015). Standards and guidelines for sequence variant interpretation. Genet. Med..

[28] Abou Tayoun A.N., Pesaran T., DiStefano M.T., Oza A., Rehm H.L., Biesecker L.G., Harrison S.M. (2018). Recommendations for interpreting loss-of-function variants (PVS1). Hum. Mutat..

[29] Cummings B.B., Karczewski K.J., Kosmicki J.A., Seaby E.G., Watts N.A., Singer-Berk M., Mudge J.M., Karjalainen J., Satterstrom F.K., O’donnell-Luria A.H. (2020). Transcript expression-aware annotation improves rare variant interpretation. Nature.

[30] Seidizadeh O., Cairo A., Oriani C., Peyvandi F. (2026). Updated global prevalence and ethnic diversity of von Willebrand disease based on population genetics analysis. Sci. Rep..

[31] Sun K.Y., Bai X., Chen S., Bao S., Zhang C., Kapoor M., Backman J., Joseph T., Maxwell E., Mitra G. (2024). A deep catalogue of protein-coding variation in 983,578 individuals. Nature.

[32] Holmberg L., Berntorp E., Donnér M., Nilsson I.M. (1986). von Willebrand’s disease characterized by increased ristocetin sensitivity and the presence of all von Willebrand factor multimers in plasma. Blood.

[33] Holmberg L., Dent J.A., Schneppenheim R., Budde U., Ware J., Ruggeri Z.M. (1993). von Willebrand factor mutation enhancing interaction with platelets in patients with normal multimeric structure. J. Clin. Investig..

[34] Baronciani L., Federici A.B., Castaman G., Punzo M., Mannucci P.M. (2008). Prevalence of type 2B ‘Malmö/New York’ von Willebrand disease in Italy: The role of von Willebrand factor gene conversion. J. Thromb. Haemost..

[35] Federici A.B., Mannucci P.M., Castaman G., Baronciani L., Bucciarelli P., Canciani M.T., Pecci A., Lenting P.J., De Groot P.G. (2009). Clinical and molecular predictors of thrombocytopenia and risk of bleeding in patients with von Willebrand disease type 2B: A cohort study of 67 patients. Blood.

[36] Ahmad F., Jan R., Kannan M., Obser T., Hassan I., Oyen F., Budde U., Saxena R., Ahmad F., Schneppenheim R. (2013). Characterisation of mutations and molecular studies of type 2 von Willebrand disease. Thromb. Haemost..

[37] Veyradier A., Boisseau P., Fressinaud E., Caron C., Ternisien C., Giraud M., Zawadzki C., Trossaert M., Itzhar-Baïkian N., Dreyfus M. (2016). A laboratory phenotype/genotype correlation of 1167 French patients from 670 families with von Willebrand disease: A new epidemiologic picture. Medicine.

[38] Yadegari H., Halimeh S., Krahforst A., Pavlova A., Pezeshkpoor B., Müller J., Pötzsch B., Biswas A., Marquardt N., Scholz U. (2026). Landscape and spectrum of VWF variants in type 2 von Willebrand disease: Insights from a German patient cohort. Thromb. Haemost..

[39] Krahforst A., Yadegari H., Pavlova A., Pezeshkpoor B., Müller J., Pötzsch B., Scholz U., Richter H., Trobisch H., Liebscher K. (2024). Unravelling the spectrum of von Willebrand factor variants in quantitative von Willebrand disease: Results from a German cohort study. J. Thromb. Haemost..

[40] McVey J.H., Rallapalli P.M., Kemball-Cook G., Hampshire D.J., Giansily-Blaizot M., Gomez K., Perkins S.J., Ludlam C.A. (2020). The European Association for Haemophilia and Allied Disorders (EAHAD) Coagulation Factor Variant Databases: Important resources for haemostasis clinicians and researchers. Haemophilia.

[41] Stenson P.D., Mort M., Ball E.V., Chapman M., Evans K., Azevedo L., Hayden M., Heywood S., Millar D.S., Phillips A.D. (2020). The Human Gene Mutation Database (HGMD): Optimizing its use in a clinical diagnostic or research setting. Hum. Genet..

[42] Desch K.C., Ozel A.B., Siemieniak D., Li J.Z., Ginsburg D. (2013). Linkage analysis identifies genetic determinants of plasma von Willebrand factor. Blood.

[43] Smith N.L., Chen M.H., Dehghan A., Strachan D.P., Basu S., Soranzo N., Hayward C., Rudan I., Sabater-Lleal M., Bis J.C. (2010). Novel associations of multiple genetic loci with plasma levels of factor VII, factor VIII, and von Willebrand factor. Circulation.

[44] Rydz N., James P.D. (2012). The evolution and value of bleeding assessment tools. J. Thromb. Haemost..

[45] Connell N.T., Flood V.H., Brignardello-Petersen R., Abdul-Kadir R., Arapshian A., Couper S., Grow J.M., Kouides P., Laffan M., Lavin M. (2021). ASH ISTH NHF WFH 2021 guidelines on the management of von Willebrand disease. Blood Adv..

[46] Tosetto A., Castaman G., Rodeghiero F. (2008). Bleeding scores in inherited bleeding disorders. Blood Rev..

[47] D’Apolito M., D’Andrea G., Palladino G.P., Ceccarini C., Cordisco G., Leccese A., Adipietro I., Stanziale P., Margaglione M., Santacroce R. (2026). Incidental findings of inherited thrombosis and hemostasis disorders in clinical exomes with unrelated indications. Haematologica.

[48] Santacroce R., D’Andrea G., Tiscia G.L., Lassandro G., D’apolito M., Barcellona D., De Bonis P., Marongiu F., Giordano P., Grandone E. (2025). Clinical and genetic heterogeneity of factor XI deficiency: Insights from a Southern Italian cohort. Int. J. Mol. Sci..

[49] Naqvi A.A.T., Alajmi M.F., Rehman T., Hussain A., Hassan I. (2019). Effects of Pro1266Leu mutation on structure and function of glycoprotein Ib binding domain of von Willebrand factor. J. Cell Biochem..

[50] Federici A.B., Canciani M.T. (2009). Clinical and laboratory versus molecular markers for a correct classification of von Willebrand disease. Haematologica.

[51] Federici A.B. (2018). How I treat type 2B von Willebrand disease. Blood.

[52] Mazurier C., Gaucher C., Jorieux S., Goudemand M. (1994). Biological effect of desmopressin in eight patients with type 2N von Willebrand disease. Br. J. Haematol..

[53] Cacheris P.M., Nichols W.C., Ginsburg D. (1991). Molecular characterization of a unique von Willebrand disease variant. J. Biol. Chem..

[54] Hilbert L., Gaucher C., Mazurier C. (1994). Identification of two mutations in the FVIII-binding domain of von Willebrand factor in type 2N disease. Blood.

[55] Mazurier C. (1992). von Willebrand disease masquerading as haemophilia A. Thromb. Haemost..

[56] Nishino M., Girma J.P., Rothschild C., Fressinaud E., Meyer D. (1989). New variant of von Willebrand disease with defective binding to factor VIII. Blood.

[57] Baronciani L., Peake I., Schneppenheim R., Goodeve A., Ahmadinejad M., Badiee Z., Baghaipour M.-R., Benitez O., Bodó I., Budde U. (2021). Molecular characterization of type 3 von Willebrand disease: 3WINTERS-IPS. Blood Adv..

[58] National Center for Biotechnology Information ClinVar: VWF Variation Records and Germline Classifications. https://www.ncbi.nlm.nih.gov/clinvar/?term=VWF%5Bgene%5D.

[59] Wagner A.H., Babb L., Alterovitz G., Baudis M., Brush M., Cameron D.L., Cline M., Griffith M., Griffith O.L., Hunt S.E. (2021). The GA4GH Variation Representation Specification: A computational framework for variation representation and federated identification. Cell Genom..

[60] (2024). Clinical Genome Resource von Willebrand Disease Variant Curation Expert Panel. Specifications to the ACMG/AMP Variant Interpretation Guidelines for VWF, Version 1.0; ClinGen Criteria Specification Registry.

