# Supplementary material for: Population Frequencies of VWD-Associated Genotypes: Cross-Resource Evidence from RGC-ME and gnomAD
Source: Int J Mol Sci. 2026 Oct 5;27(19):8866. doi: 10.3390/ijms27198866 (PMC13665229; doi:10.3390/ijms27198866)
Supplement: Supplementary file 1 [file ijms-27-08866-s001.zip › ijms-4579168-supplementary.pdf]

Supplementary Figure S1

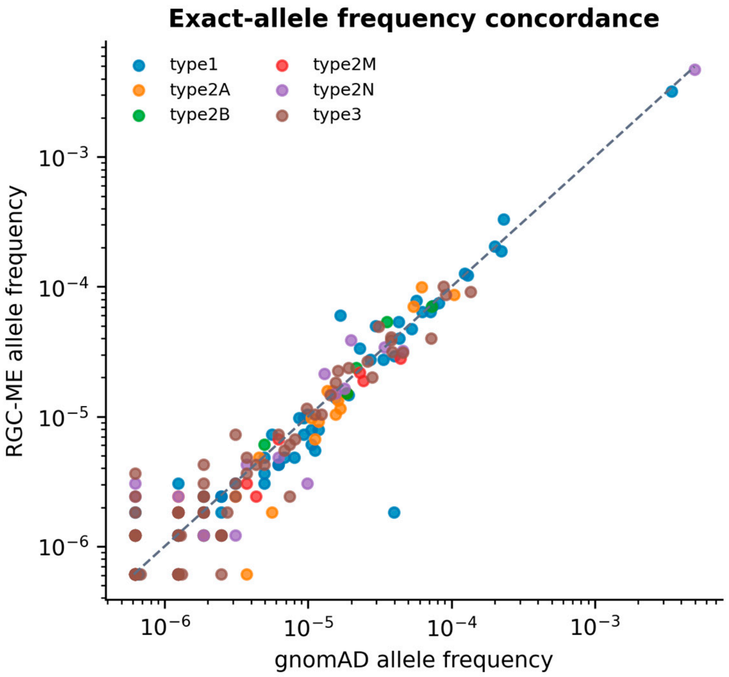

Supplementary Figure S1. Allele-frequency concordance for the 197 unique phenotype-curated alleles observed as exact GRCh38 matches in both resources. The dashed line denotes identity.
